# A Non-bipartite Propensity Score Analysis of the Effects of Teacher–Student Relationships on Adolescent Problem and Prosocial Behavior

**DOI:** 10.1007/s10964-016-0534-y

**Published:** 2016-07-05

**Authors:** Ingrid Obsuth, Aja Louise Murray, Tina Malti, Philippe Sulger, Denis Ribeaud, Manuel Eisner

**Affiliations:** 1 Institute of Criminology, University of Cambridge, Sidgwick Site, Cambridge, CB3 9DA UK; 2 Department of Psychology, University of Toronto, 100 St. George Street, 4th Floor, Sidney Smith Hall, Toronto, ON M5S 3G3 Canada; 3 Bern, Switzerland; 4 Crime Research Unit, Department of Sociology, Federal Institute of Technology, Zurich, Clausiusstrasse 59, RZ E 2, 8092 Zurich, Switzerland

**Keywords:** Teacher–student relationship, Problem behavior, Prosocial behavior, Longitudinal design, Non-bipartite matching

## Abstract

Previous research suggests a link between the quality of teacher–student relationships and the students’ behavioral outcomes; however, the observational nature of past studies makes it difficult to attribute a causal role to the quality of these relationships. In the current study, therefore, we used a propensity score analysis approach to evaluate whether students who were matched on their propensity to experience a given level of relationship quality but differed on their actual relationship quality diverged on their concurrent and subsequent problem and prosocial behavior. Student/self, teacher, and parent- (only waves 1–3) reported data from 8 waves of *the Zurich Project on the Social Development of Children and Youths (z*-*proso), a longitudinal study of Swiss youth* among a culturally diverse sample of 7- to 15-year-olds were utilized. The initial sample included *1483 (49.4* *% female) students for whom information relevant for this study was available. The sample represented families from around 80 different* countries, from across all the continents; with approximately 42 % of the female primary caregivers having been born in Switzerland. Following successful matching, we found that students who reported better relationships with their teachers and whose teachers reported better relationships with them evidenced fewer problem behaviors concurrently and up to 4 years later. There was also evidence for an analogous effect in predicting prosocial behavior. The implications of these findings are discussed in relation to prevention and intervention practices.

## Introduction

It is well known that supportive adults, other than those within the family, are of crucial importance in behavioral, social and emotional development throughout childhood and adolescence (e.g., Silver et al. 2005; Tiet et al. 2010; Troop-Gordon and Kopp 2011). For example, a recent study (Oberle et al. 2014) of 3026 fourth Graders evaluated the impact of a range of supportive relationships within the family, school and neighbourhood. The results indicated that relationships with teachers or other adults in school are the strongest predictors of emotional well-being, with children viewing school-based relationships as even more important than familial support. While there is much evidence supporting the link between teacher–student relationship quality and well-being in young children (e.g., Maldonado-Carreño and Votruba-Drzal 2011; O’Connor et al. 2011), much less is known about the concurrent link in adolescence or about the effects of earlier teacher–student relationships on adolescents. In this study, we examined the effects of the quality of teacher–student relationships assessed at age ten on problem (aggressive and oppositional) and prosocial behavior concurrently and through adolescence up to age 15.

Several developmental theories place importance on teacher–student relationships in students’ development, including attachment-based theory, socialization theory, interpersonal theory, developmental systems theory, and social-motivation theory (e.g., Sabol and Pianta 2012; Spilt et al. 2011). Each of these emphasizes somewhat different aspects of teacher–student interactions; however, all recognize the importance of emotional support, connectedness, closeness and sensitivity as key determinants of teacher–student relationship quality. Evidence suggests that relationships with these characteristics foster healthy socio-emotional development and well-being (e.g., Vesely et al. 2013). They may also protect students with higher levels of initial problem behavior from following an increasing problem behavior trajectory through adolescence (Silver et al. 2005).

While randomized controlled trials are considered the “gold standard” for identifying causal effects of teacher–student relationships, there remains an important role for ecological, observational data. This is because for dyadic variables in multi-level contexts, such as classrooms governed by the interplay of a multitude of social-contextual and individual-dispositional factors, it is far from clear how results from experimental studies generalize to the real world. The few intervention studies that have been conducted found support for the idea that teacher–student relationships impact on behavioral outcomes (e.g., Driscoll and Pianta 2010; Vancraeyveldt et al. 2015); however, they did not examine whether the change in behavior was a direct consequence of the achieved teacher–student relationship and were focussed on pre-schoolers; providing limited information about effects later in development.

Observational studies by and large also support the idea that teacher–student relationships are important for both positive and negative behavior outcomes. For instance, better teacher–student relationships have been associated with fewer anti-social behaviors (Lang et al. 2013; Silver et al. 2005; Tiet et al. 2010) and more prosocial behavior in childhood (Howes et al. 1994; Roorda et al. 2014; Wentzel 1998). Some studies have found age-limited effects, for example, Howes et al. (1994) showed that students’ relationships with their teachers at age one and two were not related to later prosocial behavior but their relationship with their teacher in preschool, at age three, was. However, the flip side of the greater ecological validity of observational data is that it creates challenges with respect to accounting for alternative explanations for an apparent causal effect of relationship quality on behavioral outcomes. Jaffee et al. (2012), for example, list four major challenges: (1) reverse causation, (2) misidentification, (3) social selection, and (4) confounding with a third variable. Reverse causation refers to the possibility that the putative outcome is really the causal factor while the putative causal factor is, in actuality, the outcome; in this case that teacher–student relationships are the outcome of, rather than cause of, behavioral outcomes. Misidentification refers to the possibility that it is not the putative causal factor itself but some correlated feature of that causal factor that is responsible for the effect. Social selection refers to the idea that individuals select and shape their exposures in a manner consistent with their dispositions. Confounding with a third variable refers to the possibility that some unmodelled factor causes both the student–teacher relationship quality and the behavioral outcomes.

Indeed, these kinds of challenges have been noted in relation to the attribution of causality in teacher–student relationships. For example, students are not randomly distributed with respect to their teacher–student relationships; rather some students are more likely than others to elicit negative relationships based on their dispositions. In particular, teacher–student relationships are likely to be adversely affected by problem behavior or poor social competence on the part of the student (e.g., Birch and Ladd 1998; Blankemeyer et al. 2002). Therefore, any association could partly or wholly reflect reverse causality. It has also been noted that a large number of characteristics of the student and their family environment may affect both the relationship with the teacher and the level of problem behavior, thus, the possibility for unmeasured confounding has also been acknowledged (Duncan et al. 2004).

Previous studies have helped to address these possibilities in several ways. First, by using longitudinal designs that repeatedly measure both teacher–student relationships and the behavioral outcome of interest over time, it has been suggested that poor quality teacher–student relationships are both a cause and effect of aggressive behavior. For example, Doumen et al. (2008) documented bi-directional cross-lagged effects of teacher–student relationships and externalizing problems over a 1-year time span in kindergarten. Theimann (2016) showed similar effects with respect to teacher–student relationships, delinquent behaviors and prosocial attitudes, although only a subset of all the tested cross-lagged paths from relationship to behavioral outcome were statistically significant. Second, if the effects of teacher–student relationships persist after controlling for a range of potentially confounding or correlated features, this helps to increase confidence that the causal factor has been correctly identified. For example, Tiet et al. (2010) provided evidence for associations between positive teacher–student relationships and fewer antisocial behaviors over and above the effects of delinquent peers, adverse life events and negative parenting. However, neither longitudinal designs nor covariate control alone guarantee that comparison groups (here those differing in levels of teacher–student relationship quality) are equivalent in all relevant respects and thus cannot fully address the above-mentioned challenges to causal inference.

One of the most powerful designs with respect to attributing causality in observational data is the propensity score model (see e.g., Jaffee et al. 2012). Propensity score analysis is based on a principle of modelling and accounting for the selection process that could lead to systematic differences between comparison groups (i.e., those differing in level of teacher–student relationship quality) and in doing so correcting for any resulting bias (Schafer and Kang 2008). The aim is to “re-balance” these groups by comparing individuals with similar propensities to experience a given level of relationship quality. If youth who are matched on propensity in this way differ on their behavioral outcomes, this is more consistent with a causal effect of relationship quality. No study to date has—to our knowledge—applied this design in the context of teacher–student relationships and behavioral outcomes in adolescence.

## The Current Study

Given the important outstanding issues of attributing causal effects to teacher–student relationships, particularly in the under-studied period of adolescence, the aim of this study was to examine the impact of the quality of teacher–student relationships on student behavioral outcomes using a propensity score design and longitudinal data that spans early to mid-adolescence.

We selected a range of relevant covariates measured prior to the assessment of the teacher–student relationship that may affect selection into treatment condition (in our case having a particular dose/quality of relationship to a new teacher) and/or the outcomes. Achieving balance on propensity depends on the availability of rich data, measured before the particular treatment might occur/is measured, ideally derived from different informants (Haviland et al. 2007).

In this study, 105 covariates from multiple informants (student, teacher and parent) collected in the first three waves (Grades 1, 2 and 3; ages 7–9), that is prior to the teacher–student relationship assessment, were included as predictors of relationship quality in ordinal logit models. The predicted values from these models were taken as the estimates of the propensity scores. Each of the covariates has been identified in previous studies as having a link to the quality of the teacher–student relationship (e.g., Drugli 2013; Jerome et al. 2009), representing a developmental risk factor associated with problem behavior (e.g., Silver et al. 2005) and/or facilitating prosocial behavior (e.g., Newton et al. 2014; Rodkin et al. 2013). Measures at ages 7, 8 and 9 were included as covariates so that matching would not only be performed on relevant characteristics in the year before the teacher assessment but also on earlier ones. Specifically, six variables measured *student and family characteristics;* 44 variables measured *student behaviors and emotions*; 18 measured *attitudes toward school and peers*; eight measured experiences of *bullying victimization and perpetration*; 12 measured *parenting practices* and three *school cohesion*; nine were academic measures; and two covariates indicated whether the student was the recipient of one or both interventions, which were part of the larger project. Crucially, one variable per informant assessed the quality of the teacher–student relationship in the teacher–student dyad during the year prior to the allocation of a new teacher to the student (i.e. in Grade 3). This means that matching, if successful, balanced the matched pairs on the quality of the teacher–student relationship with the previous teacher.

We assessed teacher–student relationships as well as the student outcomes from the perspective of the teachers as well as the students. In line with Voisin et al. (2005), we defined and operationalized the quality of the relationship as feeling (more or less) connected to the student (teacher report) and feeling (more or less) supported and treated fairly by the teacher (student report; e.g., Meschke et al. 2012). The assessed outcomes focused on two types of problem behaviors—aggressive behavior and oppositional defiant behavior (teacher-reports only) as well as prosocial behavior. The outcomes were assessed concurrently with the teacher–student measure as well as one, three and 5 years later, in order to examine concurrent as well as long-term effects. The multi-informant approach allowed for a cross-informant evaluation of the impact of the quality of teacher–student relationships (the “treatment”) on the outcomes. We hypothesized that, compared to matched students who will report to have a less supportive and fair relationship with their teacher, those who will report to have more supportive and fair relationships will engage in fewer problem behaviors and more prosocial behaviors (based on self-reports and teacher reports) concurrently and prospectively. Similarly, we expected that, compared to the matched students to whom the teachers will report feeling less connected, those to whom teachers will report feeling more connected will engage in fewer problem behaviors and more prosocial behaviors (based on self-reports and teacher reports) concurrently and prospectively. Given the paucity of previous research on this issue, we refrained from proposing specific hypotheses regarding the strength of effects depending on the informant with respect to the relationship; instead treating this question in an exploratory manner.

In addition, we examined the role of teacher gender in the link between teacher–student relationships and outcomes. Emerging findings have contradicted the traditional belief among educationalists that boys’ underachievement may, at least in part, be explained by the under-representation of male teachers limiting boys’ exposure to gendered role models in the classroom. While the teacher and student gender match does not appear to be an important predictor of student outcomes (e.g., Cho 2012; Krkovic et al. 2014; Majzub and Rais 2010; Spilt et al. 2012; Quaglia et al. 2013), teacher and student gender independently seem to matter. A common pattern of findings is that female teachers evaluate female students more favorably than male students; while male teachers do not make such differentiations (Quaglia et al. 2013), female teachers evaluate students of both genders more favorably than male teachers do and both male and female teachers evaluate their relationships with male students less favorably than with female students (e.g., Spilt et al. 2012). However, previous research on the current sample, has suggested that teacher gender does not significantly explain any of the individual differences in teachers’ tendencies to view students more or less favorably (Murray et al. 2016). Much less is known about the role of teachers’ gender in the female versus male students’ perceptions of their relationship with them and how these are related to outcomes. Given the still unclear role of teachers’ gender in teacher–student relationships and their implications for youth development, we also evaluated whether teachers’ gender affects the relation between teacher–student relationships and student behavior.

## Methods

### Participants

The data were drawn from the first eight waves of *the Zurich Project on the Social Development of Children and Youths (z*-*proso),* an ongoing longitudinal cohort study of Swiss youth with an intervention component. Fifty-six public elementary schools were randomly sampled, stratified by school size and socioeconomic background of the school district. The target student sample at the initial assessment consisted of all 1675 first Graders from these schools (Eisner and Ribeaud 2005, 2007).

Data were collected from teachers, students and their parents annually in the first three waves (W) of data collection (ages 7, 8 and 9) from Grade 1 to 3 (W1 to W3) between 2004/5 and 2006/7. Data continued to be collected annually from the teachers up to Grade 9, with the exception of Grade 8 (ages 7, 8, 9, 10, 11, 12, 13, 15; W1 to W8) in year 2013/2014 and biennially from students (ages 11, 13, and 15; W5, W7, W8). The last data collection from parents was carried out when the students were in Grade 5 (age 11; W5). In *Zurich*, the same teacher usually teaches students from Grade 1 to 3 and from Grade 4 to 6, which is the end of primary school. After Grade 6 students enter a tiered system of secondary schools.

In the present study, teacher-reported and student-reported data from each available wave were utilized. In addition, parent-reported data from the first three waves was utilized in the propensity score model. We only included cases of students in the present analyses who experienced a teacher change between ages 9 and 10 (1483 students), and for whom data were available related to the student and/or teacher-reported teacher–student relationship. The purpose of this was to minimize the possibility of results being due to the previous interactions between the student and the particular teacher with whom relationship quality was hypothesized to be causal. Students who did not experience a teacher change at that age were more likely to have special educational needs and had either completed an extended 2-year first Grade or had been retained during the first 3 years. Of the 1483 students, information about the teacher–student relationship was available from 1176 teachers and 1067 students.

At W1, the students’ age was *M* = 7.45 years (*SD* = 0.38). The retention rate from W1 to W2, when the students’ age was *M* = 8.10 (*SD* = 0.37) was 97 % for the student interviews and 96 % for the teacher assessments; from W1 to W3 (age *M* = 9.10, *SD* = 0.37), the retention rate was 96 % for the student and 94 % for the teacher assessment; for W1 to W5 (age *M* = 11.33, *SD* = 0.37), the retention rate was 83 % for student and 77 % for the teacher assessment; for W1 to W7 (age *M* = 13.67, *SD* = 0.36), the retention rate was 85 % for student and 79 % for the teacher assessment, and for W1 to W8 (age *M* = 15.44, *SD* = 0.36), the retention rate was 92 % for student and 81 % for the teacher assessment.

Of the 1067 students included in this study, 49.9 % were girls. At W1, 78 % lived with both biological parents, 17.2 % with only one parent, 3.8 % with a biological parent and another caregiver and 1.1 % with foster parents or other caregivers. As for the socioeconomic background of the primary caregiver, of the 1016 participants for whom these data were available, 20.9 % had little or no secondary education, 40.6 % completed an apprenticeship, vocational school or passed A-levels, 17.3 % had attended vocational high school, had a baccalaureate degree or advanced vocational diploma, and 15.2 % had a university degree. Further, 5.1 % of these students were in a small class requiring special educational support.

*Zurich* has a high number of immigrants and the sample was fairly representative of those (Ribeaud and Eisner 2010). Specifically, 11 % of the students were born outside of Switzerland, and in 40 % of the cases both parents were born outside of Switzerland (representing around 80 countries of origin). All contact letters and parent interviews were translated into the nine most frequently spoken foreign languages, whereas participating students and teachers were surveyed in German. Special care was taken to recruit native speakers or cross-culturally competent interviewers for the larger immigrant communities.

Two universal prevention programs were implemented as part of the study with the aim to reduce students’ externalizing problems. Findings on the short and long term effects of the interventions are reported in Malti et al. (2011) and Averdijk et al. (2016), respectively. They yielded very limited if any evidence of intervention effects. In the present study, we included participation in the two interventions as covariates.

### Procedure

In line with the legal standards in Switzerland, written informed consent was obtained from the primary caregiver at the beginning of the first wave (student age 7) and again at the beginning of the fifth wave (student age 11). At ages 13 and 15, the students provided active consent, while parents had the possibility to opt out their child. Computer-assisted 45-min-long personal interviews (CAPIs) were conducted with the students at school at ages 7 through 9. From age 11 onwards, the students completed a written questionnaire that lasted approximately 90 min. At age 11, the questionnaires were completed by the students during regular school hours, therefore incentives were not offered. At ages 13 and 15, the questionnaires were completed outside regular school hours and the students received an incentive of approximately USD 30 for participation. Parents were administered 90-min-long CAPIs in their homes and received an incentive for their participation in about the same amount. Teachers completed a paper-and-pencil student assessment form for each participating student at all eight waves. During the first three waves of data collection teachers were not compensated as their participation was mandatory due to the intervention component of the study during these waves (for details see Eisner and Ribeaud 2005, 2007). Subsequently, from wave four onwards, teachers with at least seven participants in their class received a book voucher worth approximately 50 USD as an incentive.

### Measures

Below we present the constructs and related measures which were utilized to assess the “treatment” as well as the 105 variables, which were entered into the estimation of the propensity score for the matching (see Table 1). The latter were all measured at W1, 2 and/or 3 (when the students were aged 7–9), prior to the “treatment”, which was measured at W4 (teacher) and W5 (student), when the students were 10 and 11 years old. Some of the constructs which were utilized in the estimation of the propensity scores were also utilized as outcomes based on their measurement at W4–8 (when the students were 10–15 years old).

**Table 1:** 105 covariates included in the propensity score matching

	No. of items	Student	Teacher	Parent	Total no. of items
Teacher–student relationship	1	W3	W3	W2	3
Student characteristics	6 (Student gender, special needs class, parental education, socioeconomic status, single parent home, foreign nationality status)			W1	6
Problem behavior and emotions	5 (Prosocial, aggression, ODD anxiety/depression, ADHD)	W1, 3	W1, 2, 3	W1, 3	44
	3 (Prosocial, ODD, aggression))	W2		W2	
	1 Non-aggressive problem behavior		W1, 2, 3		
Attitude toward school and peers	1 Attitude toward homework			W2	18
	1 Attitude toward school	W2,3			
	1 Gets along with peers	W3			
	4 Child’s social role (popular, unpopular, isolated, dominates others)		W1, 2, 3		
	2 Parents’ involvement in school (parent support, parent interest)		W3		
Experiences of bullying victimization and perpetration	4 × 2 (teasing, destroying property, physical violence, rejection)	W2			8
Academic measures	3 (Math, language, motivation)		W1, 2, 3		9
School cohesion	1		W1, 2, 3		3
Parenting	4 (Involvement, controlling, erratic, corporal punishment)			W1, 2, 3	12
Treatment	2 (PATHS, 3P)				2
Total					105

W1, W2, W3—waves 1, 2, 3 of data collection; corresponding to Grades 1–3; students’ age 7–9

#### Treatment Variables

##### Teacher–Student Relationship

Because the teacher–student relationship is a dyadic construct, it is important that both parties are utilized as sources of information (e.g., Murray et al. 2008). We utilized teacher-reported and student-reported information on the teacher–student relationship. We were interested in the effects of the teacher–student relationship assessed by the teachers when the students were 10 years old (at the end of Grade 4) as well as the effect of the teacher–student relationship assessed by the students when they were 11 years old (approximately midway through Grade 5) on concurrent and subsequent behaviors when the students were aged 10 (only teacher reports), 11, 12 (only teacher reports), 13 and 15. As the first teacher change occurred between Grades 3 and 4, the teacher–student relationship was assessed by the teachers approximately 1 year after the teacher change and by the students approximately one and a half years after this change. As teacher versus student reports may provide qualitatively different information about their relationship, we were interested in the effects of each. For this reason matching was completed separately based on the teacher report information about the relationship and the student report information about the relationship.

At age ten, teachers rated the following statement: “I have a good connection with this child”. Responses were recorded on a 5-point Likert scale from strongly disagree = “1” to strongly agree = “5”. The teachers’ answer to this question was utilized as a proxy for the quality of the teacher–student relationship for the purposes of this study. Information provided during the school year after the teacher change (age 10) was utilized as the teacher-reported “treatment” variable in the subsequent analyses.

At age 11, students reported about their relationship with their teacher by rating the following three statements on a 4-point Likert scale from completely untrue = “1” to completely true = “4”: “I get along with my teacher”; “The teacher is fair to me”, and “The teacher supports me”; Cronbach’s alpha was .79. A mean score of their responses to these questions was utilized in the current analyses. The score was rounded to an integer yielding again a 4-point scale which was utilized as the student-reported “treatment” variable.

Contrary to the “ordinary” propensity score matching approach that distinguishes between a “treated” and “untreated” (control) group, i.e., that makes a dichotomization, we employ a non-bipartite approach that takes into account the ordinal scale of the treatment-measures, i.e., the relationship with the teacher in (more than two) different “doses”.

#### Matching Variables and Outcomes

##### Teacher–Student Relationships

At age nine, a parallel question to the one above was administered to the teachers and included in the estimation of the propensity scores, for short and more generally “in the matching”. At age nine, students also reported about their relationship with their teachers by answering a question rated on a 4-point Likert scale ranging from not at all = “1” to very much “4”. Parents were also asked about their child’s relationship to their teacher when the students were eight years old by rating the following question: “How well does your child get along with his\her teacher?” on a 10-point Likert scale ranging from not so well = “1” to extremely well = “10”. These variables were included as covariates for matching to control for the quality of previous teacher–student relationships based on each of the informants.

##### Student and Family Characteristics

At W1, parents answered a set of questions tapping general demographic characteristics. This information was summarized into five dichotomous variables, which were utilized in the propensity score matching. Specifically, one item coded whether the families represented a single parent home = “1” versus non-single parent home = “0”. Another item coded whether both parents were born in Switzerland = “1” versus at least one parent was not born in Switzerland = “0”. A dichotomous score was also derived based on the parents’ highest level of education, at least A-levels (i.e. completed High School) = “1” versus not = “0”. Students were also classified based on whether they attended a regular class = “0” versus a small class = “1”, the latter would suggest a need for special educational help. Finally, socio-economic status was coded based on the International Socio-Economic Index of Occupational status (ISEI; Ganzeboom and Treiman 1996) for male and female primary caregivers. These were standardized to z-scores and utilized in the matching.

##### Student Behaviors and Emotions

We utilized The Social Behavior Questionnaire (SBQ; Tremblay et al. 1991) adapted for teachers (at W1–8) and parents (W1–3) to collect teacher and parent information on students’ overt aggressive behavior, oppositional defiant behavior, prosocial behavior, anxiety/depression, non-aggressive conduct problems and ADHD symptoms. Information about all six of these variables reported by both teachers and parents at W1–3 were utilized in the matching. Only teacher-reported information about three of them—prosocial behavior, overt aggression and oppositional defiant behavior at W4–8 were utilized to assess outcomes.

Teachers and parents rated each item on a 5-point Likert scale ranging from never = “1” to very often = “5”. The *overt* *aggressive behavior* mean score was derived from eleven items on proactive aggression (four items; e.g., “S/he threatens people.”, “S/he encourages other children to pick on a particular child.”), reactive aggression (three items; e.g., “S/he reacts in an aggressive manner when contradicted.”, “S/he reacts in an aggressive manner when teased.”), and physical aggression (four items; “S/he gets into fights.”, “S/he kicks, bites, hits other children.”). Cronbach’s alphas ranged from .91 to .94 with mean alpha .93 over the eight time-points of teacher measures (ages 7–15); and they were .79, .81, and .80, respectively, at the three time points of parent measures (ages 7–9). The *oppositional defiant behavior* mean score included two items (“S/he is disobedient at school.”, “S/he ignores you, when you say something.”) and tapped students’ disobedient behavior. Cronbach’s alphas ranged from .84 to .88 with a mean alpha of .86 for the teacher reports and they were .66, .70 and .73 for the parent reports. The *prosocial behavior* mean score comprised of seven items (e.g., “S/he is good at understanding other people’s feelings.”, “S/he comforts a child who is crying or upset.”) and tapped behaviors related to helping and empathic behavior. Cronbach’s alphas ranged from .90 to .92 with a mean alpha of .91 for teacher reports and they were .77, .79, and .80 for the parent reports. The *anxiety/depression* mean score for the teacher reports included seven items and for the parent report nine items (e.g., “S/he seems to be unhappy, sad, or depressed.”, “S/he is nervous, high-strung or tense.”). For use in the matching, information from the teachers was available at W1, 2 and 3, while information from parents was only available at W1 and 3. Cronbach’s alphas were .90, .90, and .91, respectively, for the teacher reports and they were .75 and .71 for the parent reports. The *non*-*aggressive conduct problems* mean score comprised of four items based on the teacher reports and five items based on the parent reports (e.g., “S/he steals outside the home.”, “S/he destroys his/her own things.”). Cronbach’s alphas were .69, .76, and .78 for the teacher reports and .55, .60, and .63 for the parent reports at the first three waves. The *ADHD symptoms* mean score for the teacher reports included eight items and for the parent reports nine items (e.g., “S/he has difficulty awaiting turn in games or groups.”, “S/he cannot settle to anything for more than a few moments.”) assessing both of symptoms of inattention and hyperactivity. For use in the matching, information from the teachers was available at W1, 2 and 3, while information from parents was only available at W1 and 3. Cronbach’s alphas were .94, .95, and .95 for the teacher reports and they were .79 and .84 for the parent reports.

The SBQ was also utilized for the students’ self-assessment of their behaviors and emotions. Different versions of the SBQ were used in the student self-assessments for the first three waves (ages 7, 8, and 9), which were utilized in the matching and for subsequent waves (ages 11, 13 and 15), which were utilized as outcomes. For administration in the first three waves/years an adapted computer-based multimedia version of the SBQ was developed and utilized to assess the student’s reports of their own overt aggressive behavior, oppositional defiant behavior, prosocial behavior, anxiety/depression, and ADHD symptoms. As these were measured prior to the assessment of the teacher–student relationship, they were included in the matching. The measure consisted of a series of 54 drawings displaying specific behaviors of a child called “Tom” or “Tina” based on the student’s gender. For each drawing the student is asked by a voice recorded on the computer whether he/she happens to do what is shown on the drawing and responds by pressing the “Yes” or “No” button at the bottom of each screen. The administration was adapted from the “Dominic Interactif” measure (Scott et al. 2006) with a demonstrated moderate to excellent reliability and validity for young students (Campbell et al. 2006). The *overt aggressive behavior* mean score was derived from twelve questions covering proactive aggression, reactive aggression, and physical aggression (e.g., “When you are mad at someone, do you sometimes say bad things behind their back, like Tom/Tina?”. Cronbach’s alphas at ages 7, 8 and 9 were .72, .72, and .73, respectively. The *oppositional defiant behavior* mean score was derived from four questions (e.g., “Do you sometimes disobey at school when the teacher asks you to do something, like Tom/Tina?”. Cronbach’s alphas at ages 7, 8 and 9 were .62, .67, and .66. The *prosocial behavior* mean score was derived from ten questions tapping prosocial emotions and behaviors (e.g., “Do you easily recognize whether somebody is happy or sad, just like Tom/Tina?”). Cronbach’s alphas at ages 7, 8 and 9 were .59, .60, and .65. The *anxiety/depression* mean score was derived from answers to nine questions (e.g., “Do you cry sometimes, just like Tom/Tina?”) tapping symptoms of anxiety and depression. These were measured twice, at ages 7 and 9 with Cronbach’s alphas .62 and .71. The *ADHD symptoms* mean score was derived based on answers to eight questions (e.g., “Do you find it difficult to wait for your turn in games or in groups, like Tom/Tina?”) tapping both inattention and hyperactivity. These were measured twice, only at ages 7 and 9 as well. Cronbach’s alphas were .58 and .64.

From age 11, a paper and pencil version of the SBQ was administered with a 5-point Likert response scale parallel to that utilized in the teacher and parent reports. Consistent with the teacher-reported outcomes, we utilized the overt aggressive behavior and prosocial scales from W4–8 as outcomes. Oppositional defiant behaviors were not included in the student-assessments at these waves. The *overt aggression* and *prosocial behavior* mean scores were comprised of parallel items to the teacher scales. Cronbach’s alphas were .76, .84, and .83 for aggressive behavior and .80, .82 and .80 for prosocial behavior at ages 11, 13 and 15.

##### Attitudes Toward School and Peers

To assess the students’ attitude toward homework at W2 parents were asked to rate the following question: “How much does <?> like to do his\her homework?” on a 10-point Likert scale ranging from not that much = “1” to extremely much = “10”. At W2 and 3, the students were asked to rate the degree to which they “like to go to school” and at W3 they were also asked “how well do [they] get along with the other kids in their classroom”. Each also on 4-point Likert scale ranging from not at all = “1” to very much “4”. In addition, at W3, the teachers were asked to rate two statements on a 5-point Likert scale ranging from strongly disagree = “1” to strongly agree = “5”. One item assessed the parents’ “interest in the students’ school career/academic development” and the other one whether the teachers are “being supported in [their] work by the student’s parents.” At W1–3, teachers also rated the degree to which each student is “popular”, “victimized”, “isolated”, and “dominating” among their peers on a 5-point Likert scale from does not apply at all = “1” to applies very much = “5”. These scores were utilized in the matching.

##### Bullying Victimization and Perpetration

At W2, students answered eight questions adapted from Olweus (1993), which asked them both about their experiences of being victims of bullying (four items; being physically attacked; ignored or excluded; insulted or taunted; and having had their belongings taken or destroyed) and engaging in bullying behaviors themselves (4 items; parallel to victimization). Each of these items were answered on a 5-point Likert scale ranging from never = “1” to (almost) daily = “5”. The reference period for this measure was “since last summer holidays”, that is an approximate span of 2–3 months. All eight items were included in the matching. This information was not collected at W1 and 3.

##### Academic Measures

At W1–3, teachers were also asked to rate each student, relative to their peers, on their performance in maths, language and their motivation. They answered each item on a 5-point Likert scale ranging from much worse = “1” to much better = “5”. These scores were utilized in the matching.

##### School Cohesion

At W1–3, teachers also answered five questions assessing school cohesion; “The students in this school: … help each other.”, “…trust each other”, “… are motivated to join school projects”, “…. get along with each other”, and “…. have a high class cohesion”. Each item was rated on a 5-point Likert scale ranging from does not apply at all = “1” to applies very much = “5”. Mean scores of *overall school cohesion* were calculated for each wave and utilized in the matching. Cronbach’s alphas were .85, .82, and .84, respectively.

##### Parenting

We utilized The Alabama Parenting Questionnaire (APQ; Shelton et al. 1996) to assess a wide range of parenting practices at W1–3. Parents rated each item on a 5-point Likert scale ranging from never = “1” to always = “5”. *The parental involvement* mean score was calculated based on ten items (e.g., “You play games or do other fun things with your child”). Cronbach’s alphas at ages 7, 8 and 9 were .63, .69, and .67. The *inconsistent discipline* mean score was calculated based on six items (e.g., “You threaten to punish your child and then do not actually punish him/her.” with Cronbach’s alphas .52, .57, and .58. The *corporal punishment* mean score was derived from three items (e.g., “You spank your child with your hand when s/he has done something wrong.”) with Cronbach’s alphas .53, .54, and .55. The *poor monitoring* mean score was derived from ten items (e.g., “Your child is out with friends you don’t know.”) with Cronbach’s alphas .64, .69, and .74. All four subscales assessed at each of the three waves (ages 7–9) were utilized in the matching.

##### Treatment Involvement

As this was a mixed design study which included a cluster-randomized trial of two universal preventive interventions (PATHS and Triple P) implemented in W1–3 (Malti et al. 2011), the students’ participation in each was also included in the matching.

### Analytical Procedure

We applied the *optimal non*-*bipartite matching technique* (Lu et al. 2011) to identify pairs of students matched on their propensities to experience given levels of teacher–student relationship quality. As mentioned above, with this approach we take into account that the ordinal scale of the measures on the relationship with the teacher has more than two different doses. All we require is that the matched pairs are different in doses and that they are similar with respect to the above illustrated covariates. In doing so, we take, in a flexible manner, advantage of the finer measurement of the relationship with the teacher. The particular algorithm that we used, described in Lu et al. (2001), matches students in order to satisfy the requirement of the minimization of the differences in the characteristics of the matched pairs while accounting also for the requirement that the matched students must experience different relationship qualities. Both criteria, the difference in doses as well as the similarity in characteristics are assessed in conjunction via the construction of a single number, or *distance measure*, that is composed of the difference in doses (denominator) and the difference in the characteristics (numerator). The latter difference is assessed by a single scalar that is the linear prediction using the estimated coefficients of an ordinal logit model with the teacher–student relationship as the dependent variable and a series of characteristics (described above) as independent variables. We will, henceforth, refer to this single scalar as the “propensity score”, or propensity to experience a particular teacher–student relationship. The *distance measures* (composed of the differences in characteristics and the difference in doses) between any pair of two students in the sample was utilized for the optimal non-bipartite matching conducted in R Core Team (2016) with the package “nbpMatching” developed by Beck et al. (2016). We required matched pairs to be within 0.15 standard deviations on the (balancing) propensity score (Snodgrasse et al. 2011). After the matching, we carried out a set of paired samples *t* tests to assess the balance for each covariate that was used to estimate the propensity score. We also utilized paired samples *t* tests to assess the differences in outcomes in the matched pairs of “treated” (more positive teacher–student relationship than the one of the matched student; higher dose of treatment) versus “untreated” (less positive teacher–student relationship than the one of the matched student; low dose of treatment). We calculated effect sizes with the R-package “effsize” for paired samples (Torchiano 2016) after having removed incomplete cases manually.

## Results

### Descriptive Analyses

Of the 1067 adolescents who provided information about their relationship to their teacher, 11 (1.0 %) reported to have a poor relationship with them, 64 (6.0 %) reported to have a somewhat poor relationship, 384 (36.0 %) reported to have a somewhat good relationship and 608 (57.0 %) reported to have a good relationship with their teacher. The correlation between the teacher-reported (*M* = 4.06, *SD* = 0.88) and student-reported (*M* = 3.49, *SD* = 0.66) quality of the teacher–student relationship was .17, which was significant at *p* < .001.

### Deriving the “Treated” Versus “Untreated/Control” Group

#### Estimation of the Score

We ran the ordinal logit model that relates the teacher–student relationship to 105 covariates and derived the propensity scores that were used subsequently in the matching to get the pairs with different doses. We used listwise deletion, therefore, the initial sample size of 1176 individuals for whom this information was available was reduced to 738. For the student-reported relationship, the initial sample size was reduced to 699.

#### Matched Pair Distribution

The matching algorithm yielded 341 matched pairs for the teacher-reported relationship. That is, 682 students entered the final analyses and 56 out of the 738 for whom we had this information were not matched. In other words, the algorithm identified 341 dyads of students, in which one student was reported to have a more “positive” relationship with their teacher (the “treated”) and the other one a less “positive” relationship (the “untreated”), but they were at the same time very similar on the 105 covariates. With respect to student-reported data, the algorithm yielded 254 matched pairs; 508 students entered the final analyses and 191 were not matched.

#### Sample Differences

To examine whether students in the final sample—those who were matched based on the teacher and/or student reported quality of relationships (*n* = 738) were different from the rest of the total sample (*n* = 937), we carried out a series of Χ^2^ and *t* tests related to demographic characteristics as well as baseline (W1; age 7) scores on the outcome variables of interest in this study. These analyses suggested that the matched sample was not significantly different from the sample of participants who remained unmatched in terms of gender (*Χ* ^2^ = .39, *p* = .555); socio-economic status (*Χ* ^2^ = 3.45, *p* = .328); or migration status (*Χ* ^2^ = .65, *p* = .448). The students that entered the matching were not significantly different based on their self-reported prosocial, aggressive or oppositional behaviors. They were, however, reported by their teachers to be more prosocial (*t* = −3.92, *p* < .001), less aggressive (*t* = 2.59, *p* = .010) and less oppositional (*t* = 2.55, *p* = .011) at age 7 than students who did not enter the matching.

#### Post-match Balance

The t-statistic indicated no significant differences between the two groups based on the 105 matching variables, which entered the balancing score (see Table 6 of “Appendix 1”). This suggests that matching was successful.

### The Effect of Teacher–Student Relationship on Student Aggressive and Prosocial Behavior Outcomes

In the next step, we utilized paired samples *t* tests to assess the differences in outcomes in the matched pairs. Results are organized by the informant providing the information on relationship quality and behavior.

#### Teacher-Reported Teacher–Student Relationship

##### Teacher-Reported Outcomes

Consistent with our hypotheses, students, who according to their teachers had a more positive relationship with them, were viewed by the teachers as engaging in more prosocial behaviors and fewer aggressive and oppositional defiant behaviors than their matches whom the teachers saw as having a less positive relationship with them. This was the case with respect to behaviors measured concurrently at age ten as well as one year later when the students were 11 years old. The effect sizes were small (Cohen’s *d* = −0.13 to 0.37) with the largest effect size for prosocial behavior at both ages and smallest for aggressive behavior at age 11. At age 12, the trend continued, as the pattern of findings remained similar, however, the difference was only significant for prosocial behavior with an effect size of 0.24, which was still larger than the effects sizes for problem behavior at any age. Similarly, at age 13, the pattern of findings remained the same, however, at this age the difference was only significant for aggressive behavior with an effect size of −0.13. No significant differences were found in teacher reported behaviors at age 15 for any of the outcomes (see Table 2).

**Table 2:** Teacher-reported teacher–student relationship and outcomes (341 matched pairs)

Student age	Outcome	“Low dose”	“High dose”		“Low dose”	“High dose”	*d*
		*M* (*SD*)	*M* (*SD*)	*t* (*n*)	*n*	*n*	
	*Teacher*-*reported*						
10	Prosocial behavior	2.03	2.40	6.89***	341	341	0.37
		(0.74)	(0.79)	(341)			
10	Aggressive behavior	0.60	0.41	−3.77***	341	341	−0.20
		(0.72)	(0.62)	(341)			
10	Oppositional defiant behavior	0.49	0.28	−3.95***	341	341	−0.21
		(0.80)	(0.63)	(341)			
11	Prosocial behavior	2.13	2.47	4.49***	280	295	0.29
		(0.77)	(0.83)	(245)			
11	Aggressive behavior	0.51	0.39	−2.03*	280	295	−0.13
		(0.65)	(0.56)	(245)			
11	Oppositional defiant behavior	0.46	0.29	−2.32*	280	295	−0.15
		(0.76)	(0.61)	(245)			
12	Prosocial behavior	2.21	2.42	3.50**	256	280	0.24
		(0.80)	(0.80)	(214)			
12	Aggressive behavior	0.48	0.40	−1.30	256	280	−0.09
		(0.67)	(0.57)	(214)			
12	Oppositional defiant behavior	0.496	0.39	−1.19	256	280	−0.08
		(0.86)	(0.73)	(214)			
13	Prosocial behavior	2.07	2.09	0.42	278	273	0.03
		(0.84)	(0.82)	(225)			
13	Aggressive behavior	0.36	0.28	−2.00*	278	273	−0.13
		(0.51)	(0.43)	(225)			
13	Oppositional defiant behavior	0.34	0.27	−1.59	278	273	−0.11
		(0.66)	(0.60)	(225)			
15	Prosocial behavior	2.07	2.11	−0.05	283	288	−0.03
		(0.79)	(0.77)	(243)			
15	Aggressive behavior	0.35	0.34	−0.15	283	288	−0.01
		(0.49)	(0.54)	(243)			
15	Oppositional defiant behavior	0.34	0.43	1.45	283	288	0.09
		(0.67)	(0.75)	(243)			
	*Student*-*reported*						
11	Prosocial behavior	3.69	3.78	1.82	307	313	0.11
		(0.71)	(0.65)	(283)			
11	Aggressive behavior	1.54	1.54	−0.28	307	313	−0.02
		(0.43)	(0.46)	(283)			
13	Prosocial behavior	3.51	3.49	−0.46	297	303	−0.03
		(0.68)	(0.69)	(266)			
13	Aggressive behavior	1.77	1.75	−0.20	297	303	−0.01
		(0.61)	(0.59)	(266)			
15	Prosocial behavior	3.53	3.58	0.80	320	319	0.05
		(0.64)	(0.64)	(299)			
15	Aggressive behavior	1.71	1.64	−1.07	320	319	−0.06
		(0.57)	(0.52)	(299)			

“High dose” students with a more positive relationship with their teacher (the “treated”); “low dose” students with a less positive relationship with their teacher (the “controls”); *t* tests are paired samples *t* tests and corresponding matched pairs which entered the analyses

* *p* < .05; ** *p* < .01; *** *p* < .001

##### Student-Reported Outcomes

Student self-reports of behaviors suggested no significant differences between matched pairs of students whose teachers reported having a less positive versus more positive relationship with them (Table 2). This was the case with respect to outcomes one year after the teacher-based assessment of the relationship, at age 11 (note that no concurrent student-reported outcome data were available for the students at age ten), as well as when the students were 13 and 15 years old.

#### Student-Reported Teacher–Student Relationship

##### Teacher-Reported Outcomes

As predicted and consistent with the teacher-reported relationship findings, students with a self-reported more positive teacher–student relationship at age 11 were seen by their teachers as engaging in more prosocial behaviors and fewer aggressive and oppositional defiant behaviors when measured concurrently at age 11 as well as one year later at age 12 (see Table 3). The effect sizes were small (*d* = −0.18 to 0.32) with the largest effect size for concurrent oppositional defiant behavior and smallest for aggressive behavior one year later.

**Table 3:** Student-reported teacher–student relationship and outcomes (254 matched pairs)

Student age	Outcome	“Low dose”	“High dose”		“Low dose”	“High dose”	*d*
		*M* (*SD*)	*M* (*SD*)	*t* (*n*)	*n*	*n*	
	*Teacher*-*reported*						
11	Prosocial behavior	2.15	2.47	3.68***	220	239	0.26
		(0.79)	(0.88)	(208)			
11	Aggressive behavior	0.59	0.36	−3.45***	220	239	−0.24
		(0.75)	(0.51)	(208)			
11	Oppositional defiant behavior	0.60	0.26	−4.66***	220	239	−0.32
		(0.87)	(0.57)	(208)			
12	Prosocial behavior	2.18	2.48	3.28***	206	226	0.24
		(0.78)	(0.85)	(185)			
12	Aggressive behavior	0.57	0.37	−2.48**	206	226	−0.18
		(0.75)	(0.56)	(185)			
12	Oppositional defiant behavior	0.65	0.32	−3.89***	206	226	−0.29
		(0.98)	(0.65)	(185)			
13	Prosocial behavior	2.06	2.07	0.50	213	212	0.04
		(0.85)	(0.81)	(178)			
13	Aggressive behavior	0.35	0.29	−1.19	213	212	−0.09
		(0.49)	(0.47)	(178)			
13	Oppositional defiant behavior	0.38	0.25	−2.01*	213	212	−0.15
		(0.71)	(0.61)	(178)			
15	Prosocial behavior	2.11	2.11	−0.58	214	219	−0.04
		(0.78)	(0.79)	(187)			
15	Aggressive behavior	0.36	0.30	−0.82	214	219	−0.06
		(0.57)	(0.44)	(187)			
15	Oppositional defiant behavior	0.41	0.32	−1.04	214	219	−0.08
		(0.73)	(0.67)	(187)			
	*Student*-*reported*						
11	Prosocial behavior	3.60	3.81	3.50***	254	254	0.22
		(0.68)	(0.67)	(254)			
11	Aggressive behavior	1.62	1.51	−2.93**	254	254	−0.18
		(0.46)	(0.45)	(254)			
13	Prosocial behavior	3.49	3.51	0.51	230	231	0.03
		(0.65)	(0.69)	(210)			
13	Aggressive behavior	1.87	1.69	−3.36***	230	231	−0.23
		(0.64)	(0.58)	(210)			
15	Prosocial behavior	3.55	3.53	−0.32	241	245	−0.02
		(0.62)	(0.66)	(235)			
15	Aggressive behavior	1.76	1.60	−3.26***	241	245	−0.21
		(0.60)	(0.51)	(235)			

“High dose” students with a more positive relationship with their teacher (the “treated”); “low dose” students with a less positive relationship with their teacher (the “controls”); *t* tests are paired samples *t* tests and corresponding matched pairs which entered the analyses

* *p* < .05; ** *p* < .01; *** *p* < .001

Furthermore, 2 years later, reports of the new teachers (following the second teacher change) at age 13, revealed a similar pattern of findings, however, the difference was only significant for oppositional behavior with an effect size of −0.15. No significant differences were found in teacher reported behaviors at age 15.

##### Student-Reported Outcomes

Consistent with teacher-reported behaviors, students who self-reported to have a more positive relationship with their teachers at age 11 also reported to engage in fewer aggressive behaviors and more prosocial behaviors at the same age with effect sizes of −0.18 and 0.22, respectively. Similarly, two and four years later, when they were 13 and 15 years old, students with a self-reported more positive relationship with their teacher-reported to engage in fewer aggressive behaviors with effect sizes of −0.23 and −0.21, respectively. The findings related to self-reported prosocial behavior at these ages were not significant.

### Does the Impact of Teacher–Student Relationship Quality Depend on Teacher Gender?

The supplementary analyses to understand possible gender effects in our models were carried out in IBM SPSS software, Version 21.0 (IBM Corp 2012). Information was available about the teachers’ gender for 670 of the 682 students who were matched based on their teacher-reported relationship quality. Of those, 220 were male teachers and 450 were female teachers. In students where female teachers reported about their relationship, better teacher-reported relationship quality was associated with being rated by their teacher as concurrently more prosocial (*t* = 5.91, *p* < .001), less aggressive (*t* = −3.56, *p* = .001), as well as less oppositional (*t* = −3.45, *p* = .001). They were also rated as more prosocial at ages 11 (*t* = 4.13, *p* ≤ .001) and 12 (*t* = 3.51, *p* = .001), as well as less oppositional at ages 11 (*t* = −2.46, *p* = .015) and 13 (*t* = −.04, *p* = .014), and less aggressive at ages 12 (*t* = −2.10, *p* = .038) and 13 (*t* = −3.80, *p* < .001). Students also reported to engage in less aggressive behavior at ages 13 (*t* = −2.77, *p* = .007) and 15 (*t* = −3.31, *p* = .001). In students with male teachers, better teacher-reported relationship quality was associated with concurrent self-reported prosociality only (*t* = 2.22, *p* = .037). However, it should be noted that the sample size was much smaller when looking at analyses based on male teachers (ranging from 14 to 28 at different ages) compared to female teachers (ranging from 94 to 139).

Teachers’ gender information was available for 452 teachers of the 508 students that were matched based on their self-reported teacher–student relationship quality (172 male, 280 female teachers). In students with female teachers, better student-reported relationship quality was associated with teacher-reported less oppositional behavior at ages 11 (*t* = −2.80, *p* = .007) and 12 (*t* = −2.11, *p* = .038) as well as less self-reported aggression at age 15 (*t* = −2.05, *p* = .044). It was also associated with being rated by the teachers as more prosocial at age 11 (*t* = 2.08, *p* = .042). In those with male teachers, better student-reported relationship quality was associated with teacher reported more prosocial behavior at ages 11 (*t* = 2.43, *p* = .021) and 12 (*t* = 2.31, *p* = .029), as well as with less oppositional (*t* = −2.28, *p* = .030) and aggressive behaviors (*t* = −2.62, *p* = .014) at age 11. None of the remaining differences were significant. Here again the sample sizes were much smaller for the latter analyses (ranging from 29 to 19), compared to analyses related to female teachers (ranging from 74 to 54).

#### Propensity Score Matching with Teacher Gender Included

To understand the role of teacher gender in our models, we re-ran the propensity score matching procedure based on the 105 covariates plus teachers’ gender; hence 106 covariates. In doing so, the matched pairs were also required to be similar with respect to the gender of the teachers. The matching algorithm yielded 334 matched pairs for the teacher-reported relationship and 212 matched pairs for the student-reported relationship. Again, the t-statistic indicated no significant differences between the two groups based on the 106 pre-treatment (or pre-teacher assessment) characteristics, which were used in the estimation of the propensity (or balancing) score (see Table 7 of “Appendix 2”). This suggests that matching with teachers’ gender included was successful. Furthermore, examination of the effects of the quality of the teacher–student relationships on concurrent and prospective outcomes revealed the same pattern of findings as reported based on the matching without teachers’ gender (see Tables 4, 5). Furthermore, descriptive analyses and Chi square difference tests suggested no significant differences for the rate of matched pairs versus male/female student/teacher gender mixes among male versus female students. Overall, these findings suggest that the quality of the teacher–student relationship matters over and above the teachers’ gender (or the students’ gender) in relation to behavioral outcomes.

**Table 4:** Teacher-reported teacher–student relationship with teacher gender as an additional matching variable and outcomes (334 matched pairs)

Student age	Outcome	“Low dose”	“High dose”		“Low dose”	“High dose”	*d*
		*M* (*SD*)	*M* (*SD*)	*t* (*n*)	*n*	*n*	
	*Teacher-reported*						
10	Prosocial behavior	2.02	2.418	7.20***	334	334	0.39
		(0.74)	(0.78)	(334)			
10	Aggressive behavior	0.64	0.38	−5.00***	334	334	−0.27
		(0.75)	(0.56)	(334)			
10	Oppositional defiant behavior	0.50	0.27	−4.54***	334	334	−0.25
		(0.81)	(0.60)	(334)			
11	Prosocial behavior	2.12	2.50	5.14***	279	284	0.33
		(0.79)	(0.82)	(238)			
11	Aggressive behavior	0.53	0.37	−2.85**	279	284	−0.18
		(0.67)	(0.52)	(238)			
11	Oppositional defiant behavior	0.49	0.28	−3.07**	279	284	−0.20
		(0.78)	(0.57)	(238)			
12	Prosocial behavior	2.20	2.45	3.74***	258	266	0.26
		(0.81)	(0.77)	(208)			
12	Aggressive behavior	0.49	0.38	−1.46	258	266	−0.10
		(0.67)	(0.57)	(208)			
12	Oppositional defiant behavior	0.49	0.38	−1.41	258	266	−0.10
		(0.87)	(0.72)	(208)			
13	Prosocial behavior	2.10	2.10	−0.16	266	273	−0.01
		(0.83)	(0.85)	(215)			
13	Aggressive behavior	0.36	0.28	−1.15	266	273	−0.08
		(0.51)	(0.42)	(215)			
13	Oppositional defiant behavior	0.37	0.25	−1.33	266	273	−0.09
		(0.70)	(0.55)	(215)			
15	Prosocial behavior	2.12	2.07	−1.27	271	286	−0.08
		(0.81)	(0.76)	(236)			
15	Aggressive behavior	0.37	0.33	−0.38	271	286	−0.02
		(0.51)	(0.53)	(236)			
15	Oppositional defiant behavior	0.38	0.38	0.28	271	286	0.02
		(0.68)	(0.73)	(236)			
	*Student*-*reported*						
11	Prosocial behavior	3.73	3.73	−0.23	306	303	−0.01
		(0.67)	(0.70)	(279)			
11	Aggressive behavior	1.55	1.53	−0.87	306	303	−0.05
		(0.47)	(0.44)	(279)			
13	Prosocial behavior	3.51	3.48	−0.77	289	295	−0.05
		(0.65)	(0.73)	(255)			
13	Aggressive behavior	1.78	1.74	−0.82	289	295	−0.05
		(0.64)	(0.57)	(255)			
15	Prosocial behavior	3.55	3.57	0.15	310	316	0.01
		(0.61)	(0.67)	(292)			
15	Aggressive behavior	1.71	1.64	−1.38	310	316	−0.08
		(0.59)	(0.51)	(292)			

“High dose” students with a more positive relationship with their teacher (the “treated”); “low dose” students with a less positive relationship with their teacher (the “controls”); *t* tests are paired samples *t* tests and corresponding matched pairs which entered the analyses

** *p* < .01; *** *p* < .001

**Table 5:** Student-reported teacher–student relationship with teacher gender as an additional matching variable and outcomes (212 matched pairs)

Student age	Outcome	“Low dose”	“High dose”		“Low dose”	“High dose”	*d*
		*M* (*SD*)	*M* (*SD*)	*t* (*n*)	*n*	*n*	
	*Teacher*-*reported*						
11	Prosocial behavior	2.14	2.43	3.94***	212	212	0.27
		(0.80)	(0.82)	(212)			
11	Aggressive behavior	0.59	0.33	−4.61***	212	212	−0.32
		(0.75)	(0.42)	(212)			
11	Oppositional defiant behavior	0.54	0.24	−4.64***	212	212	−0.32
		(0.82)	(0.56)	(212)			
12	Prosocial behavior	2.15	2.42	3.97***	190	199	0.30
		(0.80)	(0.81)	(180)			
12	Aggressive behavior	0.57	0.33	−3.96***	190	199	−0.30
		(0.76)	(0.48)	(180)			
12	Oppositional defiant behavior	0.58	0.31	−3.69***	190	199	−0.27
		(0.96)	(0.62)	(180)			
13	Prosocial behavior	2.00	2.02	0.42	181	176	0.03
		(0.84)	(0.83)	(153)			
13	Aggressive behavior	0.36	0.29	−1.54	181	176	−0.12
		(0.49)	(0.44)	(153)			
13	Oppositional defiant behavior	0.39	0.24	−2.60**	181	176	−0.21
		(0.72)	(0.56)	(153)			
15	Prosocial behavior	2.06	2.11	1.49	178	178	0.12
		(0.76)	(0.79)	(151)			
15	Aggressive behavior	0.40	0.30	−1.34	178	178	−0.11
		(0.60)	(0.49)	(151)			
15	Oppositional defiant behavior	0.43	0.35	−0.85	178	178	−0.07
		(0.76)	(0.75)	(151)			
	*Student*-*reported*						
11	Prosocial behavior	3.57	3.76	2.71**	212	212	0.19
		(0.67)	(0.71)	(212)			
11	Aggressive behavior	1.64	1.50	−3.32***	212	212	−0.23
		(0.48)	(0.44)	(212)			
13	Prosocial behavior	3.49	3.52	0.51	195	192	0.04
		(0.67)	(0.68)	(177)			
13	Aggressive behavior	1.85	1.69	−3.49***	195	192	−0.26
		(0.64)	(0.59)	(177)			
15	Prosocial behavior	3.57	3.50	−1.27	204	199	−0.09
		(0.60)	(0.65)	(192)			
15	Aggressive behavior	1.71	1.61	−2.19**	204	199	−0.16
		(0.60)	(0.46)	(192)			

“High dose” student with a more positive relationship with their teacher (the “treated”); “low dose” students with a less positive relationship with their teacher (the “controls”); *t* tests are paired samples *t* tests and corresponding matched pairs which entered the analyses

** *p* < .01; *** *p* < .001

### Sensitivity Analyses

We conducted a number of additional analyses to assess the sensitivity of our conclusions to key methodological decisions and assess the robustness of our findings. Specifically, we carried out “ordinary” (bipartite) propensity score matching analyses in Stata 12.1 (StataCorp 2011) using the “psmatch2” function, in which we utilized common-support, no replacement and a caliper of 0.05. For the dichotomization of the teacher–student relationship variables, we set the treatment dummy for the teacher-reported relationship equal to one if the dose was larger than 3, and for the student-reported relationship if it was larger than 2. The ordinary propensity score analyses were run on parallel models to those presented above based on non-bipartite analyses—on teacher versus student reported relationships. The patterns of results with respect to the average treatment on the treated were consistent with those reported based on the primary—non-bipartite propensity score matching—analyses. Balance was also achieved, with the exception of one variable for which balance was not achieved in the matching based on the student-reported relationship. We did not investigate other (numerously) possible specifications of the matching-specifications (i.e., other definitions of the options such as, for instance, another caliper).

## Discussion

In recent years, teacher–student relationships have received sizable attention as both a source of protection, when positive, and risk when negative in relation to a wide range of student outcomes (e.g., Oberle et al. 2014; Troop-Gordon and Kopp 2011); much less is known about the effects of these relationships in adolescence. Similarly, while a handful of prevention programs improving teacher–student relationships have been developed and successfully implemented in preschools (Driscoll and Pianta 2010; Vancraeyveldt et al. 2015), none, to our knowledge, have been developed for teachers of adolescents. The lack of efforts in this area maybe due, at least in part, to lack of direct evidence for the *causal* effects of these relationships on student outcomes. While anecdotal reports suggest a general understanding of the important role of teacher–student relationships well into adolescence, current school practices suggest otherwise. Instead of fostering teacher–student relationships, providing students a sense of inclusion and belonging, schools may rely on exclusionary practices and other punitive sanctions to manage student behavior particularly with those students most at need of extra support due to a wide range of family and individual problems (e.g., Losen et al. 2015; Obsuth et al. 2016). Thus, demonstrating the causal influence of teacher–student relationships on student outcomes is both crucial and timely.

Our ability to draw conclusions that we argue can go beyond mere association, the current study owes to the use of a propensity score matching approach. This allowed us to match groups of students who developed a less versus more positive relationship with their new teachers on a wide range of characteristics measured over three years prior to the assessment of the teacher–student relationship. We were able to use both teacher and student-reported information on the quality of teacher–student relationships and behavior outcomes. By using this multi-informant propensity score matching approach, we can be more confident that our results reflect the hypothesized causal influence of teacher–student relationship than previous studies that have relied on the inclusion of covariates to control for confounding.

Teachers who reported having a more positive relationship with a student at age ten observed significantly fewer aggressive and defiant behaviors and more prosocial behaviors in the same student concurrently and one year later, at age 11. This was also associated with more prosocial behaviors two years later, at age 12 and also with less aggressive behavior at age 13. Similarly, students who perceived a more positive relationship with their teacher at age 11 reported fewer aggressive behaviors and more prosocial behaviors concurrently and also fewer aggressive behaviors two and four years later, at ages 13 and 15. When students reported a more positive relationship with their teacher, their teachers observed fewer aggressive and defiant behaviors and more prosocial behaviors concurrently, at age 11 as well as one year later at age 12. Two years later, at age 13, the students’ teachers reported fewer defiant behaviors in these students. Importantly, the effect of the quality of teacher–student relationships on behavioral outcomes was observed while matching groups on a wide range of possible alternative influences on the outcomes, including the students’ past positive or negative behavior, other mental health problems, gender, socio-economic status, experiences of bullying and/or victimization, attitudes toward school and peers, academic outcomes, or parenting practices. While the effect sizes were small (maximum 0.37), they were often comparable or larger than reported in evaluations of established school prevention programs on aggressive behavior (see e.g., Wilson et al. 2003; Wilson and Lipsey 2007). For example, the meta-analysis by Wilson and Lipsey (2007) found largest effects for interventions targeting at risk youth (around 0.41) and smallest effects for students representing the general population, comparable to our sample (around 0.09).

The results reported here build on findings by prior research (e.g., Troop-Gordon and Kopp 2011) and suggest that teacher–student relationships can causally affect a range of behaviors including aggressive behavior against peers, defiant behavior against teachers, and the lack of prosocial behavior in interaction with peers. They also show that effects on behavior problems can be found when considering the students’ perception of the relationship quality as well as when considering the teachers’ perception of the relationship. Together, these findings support the view that teacher–student relationships play a crucial role in students’ behavioral adaptation (e.g., Pianta et al. 1997; Verschueren 2015). While some previous research (e.g., Jerome et al. 2009) reported possible effects of early teacher–student relationships on behavior over up to 8 years, our findings did not consistently support long-term effects beyond one year. Specifically, teachers who reported having a more positive relationship with specific students at age 10 only reported observing more prosocial behaviors but not fewer problem behaviors two years later, when the students were 12 years old. On the other hand, at age 13, the teachers only reported observing less aggressive behaviors, but not less oppositional or more prosocial behaviors. Furthermore, no differences at all were observed by teachers five years following the assessment of the relationship. Similarly, students who rated their relationship with their teacher more positively at age 11, two and four years later, at age 13 and 15, reported engaging in less aggressive behavior but there was no effect on prosocial behaviors at these times. Consistent with this, while the new teachers reported observing fewer oppositional behaviors in these students at age 13, they did not report less aggressive or more prosocial behaviors. By age 15 these teachers reported no significant differences.

There are a few potential explanations of why teacher–student relationships may not be consistently predictive of student outcomes in early and mid-adolescence. Namely, during adolescence, while relationships with close adults remain important, peers take on a central role in adolescents’ lives and further socio-emotional development and adjustment (e.g., Blakemore and Mills 2014). In fact, the role of peer rejection has been identified as having an important impact on the development of both externalizing (Asher and McDonald 2009) and prosocial behaviors (Zimmer-Gembeck et al. 2013). While we controlled for the influence of peers prior to the teacher change, students may have, very plausibly, developed new peer relationships in the time between the teacher change, teacher–student relationship assessment and the 2–5 years follow-up. Thus, experiences with peers (e.g., peer rejection) following the teacher change, may at least partially explain the non-significant effects of teacher–student relationships on selected outcomes 2–5 years after its assessment, when the students were 12, 13 and 15. Our findings also seem to suggest, however, that there is continuing hope to shape positive outcomes; that a bad relationship with teachers does not condemn a student to poor outcomes on the long run even if they do seem to have some negative effects over the immediately following years.

Consistent with previous reports, this study revealed a significant but small correlation between the teacher- and student-reported teacher–student relationships. We administered somewhat different measures of teacher–student relationships to teachers and students, which may have attenuated concordance between the two informants. However, the majority of findings suggested consistent cross-informant results particularly with respect to the student-reported teacher–student relationship, which revealed short- and long-term positive effects based on not only student self-reports but also teacher-reported outcomes. Yet, when the teacher-reported teacher–student relationship was examined in relation to student-reported outcomes, none of these were significant. One possibility is that teacher ratings of teacher–student relationships and student behavior are subject to halo effects, whereby the latter is artificially rated as more in-keeping with the former than is merited based on actual behavior. That is, ratings of behavior are (positively or negatively) colored by the teachers’ perceived quality of their relationship with the student. However, the inconsistency may also be due to the fact that the questions in the teacher assessment and the student report were different. Either way, the question merits further exploration because whether teachers feel they have a good relationship with a student, or whether the students feel they are getting along with the teacher, may carry different implications for self-perceived and teacher-perceived behaviors. While limited evidence provides some support for this interpretation in kindergarten-age students (Murray et al. 2008), due to the paucity of research exploring these cross-informant effects, it will be important to explore this plausibility in future research.

Nonetheless, we would argue that how the student perceives their relationship with the teacher is more important with respect to their behavior than how their teacher perceives it because it is the student who engages in their behavior and thus their motivations that ultimately matter. There is some evidence for this claim in our results. Notably, there were no differences in student-reported outcomes for students differing on teacher-reported teacher–student relationship quality. Yet, students who saw themselves as having a more positive relationship with their teacher reported engaging in fewer aggressive behaviors up to age 15. This is perhaps not surprising, as it is the student’s perspective of the teacher–student relationship that likely most directly affects his or her behaviors. It is also possible, however, that the students’ perception of their relationship with the teacher was influenced by their own previous behavior more than the teachers’ perception. Moreover, it is possible that the instrument for assessing students’ perception of the relationship was more reliable than the instrument for assessing teachers’ perception. Additional cross-informant research as well as third party observational data related to the quality of teacher–student relationships may further differentiate whether a mere perception of the student of having a good relationship with the teacher or an objectively good relationship with the teacher is necessary to achieve positive student outcomes.

Overall, the results are in line with developmental theories that stress the role of healthy adult-student relationships in positive youth development (e.g., Erikson 1968; Hinde and Groebel 1991). Who the key “others” are expands throughout the lifespan. These relationships appear to influence both problem as well as prosocial behaviors in the expected direction. Adolescents with strong relationships to authority figures may be more likely to talk to them and rely on them to solve their conflict as opposed to relying on antisocial problem resolutions. These students may also be more likely to engage in prosocial behaviors via their interactions with their teachers and/or other adult authority figures, to whom they can look up to and may view them as role models. Our findings are consistent with the significant body of research (e.g., Catalano et al. 2004) guided by the Social Development Model (Hawkins et al. 1992) of behavior and behavior change, which suggests that bonds with prosocial others (peers, teachers, institutions) are a protective factor against engaging in problem behaviors. According to this model, when students develop close attachments/bonds to their teachers (and school) who promote standards for positive behavior, they are motivated to behave in a prosocial manner, consistent with the teachers’ (schools’) standards and values (Hawkins et al. 1999; Chapman et al. 2013; Voisin et al. 2005).

With respect to problem behavior, we included both aggressive behavior and oppositional defiant behavior as relevant antisocial behaviors. The pattern of findings was consistent for both of these types of behaviors suggesting that the impact of the quality of teacher–student relationships on antisocial behavior can be generalized to more than just aggressive behavior. This finding suggests a need to a focus on developing healthy, supportive and inclusive teacher–student relationships. Ideally, building healthy and supportive teacher–student relationships would become part of the curriculum in teacher training programs. Intervention programs focusing on enhancing teacher–student relationships with the aim to reduce aggressive as well as oppositional behaviors in adolescence could also be developed. To build healthy teacher–student relationships, in line with attachment theory, such interventions would focus on enabling teachers to interact with their students such that they would feel safe, secure, understood, supported and included in the school environment, which in turn would lead to fewer behavior problems, more prosocial behaviors and overall adolescent well-being (Theimann 2016; Voisin et al. 2005).

Our study also explored the role of students’ and teachers’ gender in the link between teacher–student relationships and outcomes. While we matched our two groups on students’ gender, initially we did not match the groups based on the teachers’ gender. This enabled us to examine the role of gender in the link between the teacher–student relationships and assessed behaviors. First, post hoc analyses pointed to some gender differences in the link between the quality of teacher–student relationships and student outcomes, such that, when looking at teacher-reported relationship quality, the links were only significant (with one exception of effects on prosocial behavior concurrently) where female teachers were concerned. However, this was not the case when looking at the student-reported quality of relationships. While we cannot be sure that this result did not simply reflect the smaller number of male teachers, it provides an interesting observation to further explore in future research. Second, supplementary analyses including teachers’ gender as an additional variable in the estimation of the propensity score supported our main findings and suggested that the quality of the teacher–student relationship matters over and above the teachers’ gender (as well as the students’ gender), particularly in relation to short term behavioral outcomes.

Several limitations should be noted. Utilizing an existing data set, we relied on available questionnaire data to assess teacher–student relationships in this study. Thus, while this study offers insight into the potential causal effects of teacher–student relationships, it will be important to replicate the current findings utilizing established, widely used and reliable assessment tools of teacher–student relationships (for example, the Student–Teacher Relationship Scale; Pianta and Steinberg 1992). In particular, it is possible, that in using only brief measures of relationship quality, our study underestimated the importance of relationship quality owing to the attenuation of associations due to the lesser reliability of these measures. In this study, we relied on the teacher change as a naturally occurring quasi-experimental situation, or a quasi-random assignment to a teacher with whom a student develops a more positive relationship. Teachers reported about their relationship with each student approximately one school year or 10 months after the teacher change and the students approximately one and a half school years after the teacher change. This has advantages and disadvantages. On the one hand, it allowed the teachers and students to develop a relationship that they could reliably assess. On the other hand, the amount of time between the teacher change and the relationship assessment opens the door for unmeasured differences to develop. Thus, we cannot exclude that differences occurred between the two groups of students between the time of the change and the teacher–student assessment although the two groups were very close to each other with respect to 105 (or 106 with teacher gender) covariates during the period of time prior to the change of the teacher. In general, is it not possible to rule out the possibility of unmeasured confounds though the inclusion of such a large number of relevant covariates makes this less likely. Finally, our analysis sample tended to slightly under-represent the students with the most problematic behavior at baseline. This is a common problem in observational research where individuals with the highest levels of “maladaptive” or psychopathological traits with negative social connotations are the least likely to participate and the most likely to drop-out (e.g., Kessler et al. 2005; Merikangas et al. 2010). The two most important effects of this are possible slight underestimates of the effect of relationship quality due to range restriction (e.g., Sackett and Yang 2000) and a potential lack of generalizability of our results to the students exhibiting the most problematic behavior.

Despite these caveats, this study contributes to the literature on teacher–student relationships and student mal/adaptive behaviors in several important ways. Firstly, we relied on information provided by multiple informants and explored the link between these relationships and behaviors utilizing a propensity score matching approach. This approach allowed us to match individuals on their propensity to experience a given level of relationship quality and in doing so emulate the situation of a randomized controlled trial in ecologically valid data. As a result, we are more able to conclude that the teacher–student relationship, at least for up to two years based on teacher reports and four years based on student-self reports, following the assessment of this relationship, exert what appears to be a causal influence on students’ behaviors, both positive and negative. Moreover, by applying a non-bipartite matching—as opposed to similar methodologies that allow exposure to be measured only as a binary variable—we utilized a broader range of the information related to the teacher–student relationships provided to us by teachers and students. This approach allows considering the whole information from the ordinal scale with more than two elements we have at hand for the evaluations of the relationship with the teacher. Finally, we examined the directional link between teacher–student relationships and outcomes across a span of five years and to isolate this link we utilized information spanning additional three years of the students’ lives. Thus, in total the study is based on information spanning nine years of students’ lives. Although we still do not know what the specific mechanisms are through which teacher–student relationships are related to behavioral outcomes, and this presents a crucial next step, this study is an important step in exploring this link as it suggests the possibility of a causal relationship beyond selection effects.

## Conclusion

This study shows that the quality of teacher–student relationships has the power to influence students’ behavior, both positive and negative, well into adolescence. This is the case while matching groups on a score accounting for a wide range of different factors (105 covariates, including past behaviors, parenting, school experiences etc.) that have previously been shown to be related to behavioral outcomes. These relationships appear to have a lasting effect (up to four years), which is most pronounced when students themselves see their relationship with the teachers more positively, when they feel supported by them. The effects that these relationships exert on student behaviors are stronger or comparable to those reported by findings from established school based interventions (see e.g., Wilson and Lipsey 2007). They suggest that fostering teacher–student relationships, much like fostering parent–child relationships, continues to have importance for outcomes not just in childhood but well into adolescence. Educational and school policies could take this into consideration when supporting teachers in fostering their relationships with students.

## Appendix 1

See Table 6.

**Table 6:** Post-match assessment of the balance on 105 variables between the groups of students with a “more positive” (“high dose”/“treated”) versus “less positive” (“low dose”/“controls”) relationship with their teachers based on teacher and student reports of the relationship

	Teacher–student relationship					
	Teacher-reported			Student-reported		
	*t*	*p*	SMD	*t*	*p*	SMD
Student gender	−0.16	.873	−1.17	−0.37	.711	−3.16
Foreign born status	−0.09	.928	−0.60	−0.20	.845	−1.68
Single parent home	0.90	.366	7.04	−0.58	.559	−5.08
Parental education	−0.66	.509	−4.70	−0.09	.925	−0.79
Special education	−0.18	.858	−1.33	−0.24	.809	−2.19
Socio-economic status	−0.06	.949	−0.41	0.54	.591	4.86
Received PATHS	0.68	.497	5.27	−0.17	.864	−1.57
Received Triple P	0.00	1	0.00	0.47	.639	4.03
*Student reports*						
S1 Prosocial behavior	−0.54	.588	−4.11	−0.87	.383	−8.01
S1 Anxiety/depression	−0.54	.590	−4.20	−0.83	.408	−6.78
S1 ADHD symptoms	0.19	.848	1.57	−0.42	.675	−3.64
S1 ODD behavior	0.36	.719	2.78	−0.85	.394	−7.36
S1 Aggressive behavior	0.89	.374	7.26	0.17	.865	1.42
S2 Prosocial behavior	−0.56	.575	−4.28	−0.17	.866	−1.56
S2 ODD behaviors	0.51	.611	3.93	−0.31	.759	−2.77
S2 Aggressive behavior	0.20	.846	1.55	0.38	.705	3.26
S3 Prosocial behavior	0.03	.978	0.22	0.09	.930	0.84
S3 Anxiety/depression	−0.94	.346	−7.17	0.06	.952	0.53
S3 ADHD symptoms	0.00	1	0.00	−0.12	.902	−0.99
S3 ODD behavior	0.91	.366	7.02	−0.39	.700	−3.34
S3 Aggressive behavior	0.02	.984	0.16	0.55	.581	4.66
S2 Bull Vict—teasing	−0.75	.452	−5.73	0.55	.581	4.87
S2 Bull Vict—steal/destroy	0.32	.753	2.43	−0.43	.667	−3.81
S2 Bull Vict—physical	0.31	.758	2.23	−0.55	.586	−4.93
S2 Bull Vict—rejection	0.61	.540	4.72	0.13	.899	1.14
S2 Bull Perp—teasing	−0.41	.686	−2.99	0.09	.925	0.83
S2 Bull Perp—steal/destroy	0.42	.679	3.02	−0.44	.661	−4.00
S2 Bull Perp—physical	0.24	.810	1.72	−0.59	.557	−5.18
S2 Bull Perp—rejection	0.10	.919	0.75	0.34	.735	2.92
S2 Likes school	0.00	1	0.00	0.34	.735	2.91
S3 Likes school	0.74	.459	5.71	−0.15	.879	−1.25
S3 Getting along with peers	−0.75	.456	−5.31	−0.46	.645	−3.85
S3 Teacher–student relationship	0.06	.949	0.47	−0.47	.642	−4.25
*Teacher reports*						
T1 Prosocial behavior	0.16	.874	1.16	0.62	.538	5.45
T1 Anxiety/depression	0.97	.333	7.46	−0.45	.656	−3.79
T1 ADHD symptoms	0.04	.965	0.32	0.26	.793	2.30
T1 ODD behavior	0.38	.705	2.87	−0.42	.677	−3.59
T1 Aggressive behavior	0.67	.502	4.91	0.03	.976	0.27
T1 Non-aggressive problem behavior	−0.01	.995	−0.05	−0.36	.721	−3.12
T2 Prosocial behavior	−0.43	.669	−3.11	0.37	.716	3.11
T2 Anxiety/depression	1.01	.312	7.82	−0.69	.492	−6.16
T2 ADHD symptoms	0.14	.889	1.06	0.10	.924	0.82
T2 ODD behavior	0.57	.567	4.44	−1.95	.052	−16.12
T2 Aggressive behavior	0.55	.581	4.21	−0.83	.410	−6.95
T2 Non-aggressive problem behavior	0.47	.636	3.65	−1.44	.152	−12.01
T3 Prosocial behavior	−0.52	.602	−3.88	0.68	.495	5.90
T3 Anxiety/depression	1.40	.163	10.53	−0.80	.427	−6.70
T3 ADHD symptoms	0.75	.457	5.47	−0.42	.672	−3.42
T3 ODD behavior	0.55	.580	4.25	−0.10	.919	−0.84
T3 Aggressive behavior	1.08	.280	8.53	0.46	.645	3.89
T3 Non-aggressive problem behavior	0.62	.536	4.64	−0.38	.708	−3.08
T1 Academic—math	−0.22	.827	−1.67	−0.57	.568	−5.01
T1 Academic—language	0.67	.501	5.19	0.34	.734	3.19
T1 Academic—motivation	−0.08	.935	−0.63	0.24	.812	2.14
T1 Child’s social role—popular	0.52	.604	3.72	0.09	.926	0.82
T1 Child’s social role—rejected	0.00	1	0.00	0.45	.652	3.87
T1 Child’s social role—isolated	0.05	.963	0.35	0.78	.436	6.87
T1 Child’s social role—dominant	−0.61	.545	−4.60	0.34	.737	2.97
T2 Academic—math	−0.04	.970	−0.29	−0.54	.592	−4.70
T2 Academic—language	−0.61	.544	−4.56	−0.33	.743	−3.03
T2 Academic—motivation	−0.89	.372	−6.41	0.10	.921	0.92
T2 Child’s social role—popular	−0.26	.797	−1.96	−0.05	.961	−0.44
T2 Child’s social role—rejected	0.50	.621	3.71	−0.78	.437	−7.15
T2 Child’s social role—isolated	−1.10	.272	−8.55	0.00	1	0.00
T2 Child’s social role—dominant	−0.99	.322	−7.67	−0.25	.807	−2.18
T3 Academic—math	−0.33	.744	−2.45	−0.09	.932	−0.73
T3 Academic—language	−0.39	.699	−3.05	−0.32	.753	−2.68
T3 Academic—motivation	−0.15	.885	−1.05	0.52	.604	4.65
T3 Child’s social role—popular	−0.20	.839	−1.53	0.52	.605	4.62
T3 Child’s social role—rejected	0.44	.664	3.17	0.17	.863	1.56
T3 Child’s social role—isolated	−0.25	.807	−1.85	0.53	.596	4.65
T3 Child’s social role—dominant	0.33	.744	2.46	0.16	.870	1.38
T3 Parent interested in school	−0.42	.678	−2.99	0.42	.678	3.49
T3 Parent supports school	−0.81	.417	−5.61	−0.05	.962	−0.42
T3 Teacher–student relationship	−0.99	.323	−6.60	0.17	.865	1.46
T1 School cohesion	0.44	.664	3.13	0.41	.686	3.55
T2 School cohesion	−0.08	.939	−0.52	0.72	.470	6.64
T3 School cohesion	−0.64	.524	−4.81	0.67	.507	5.84
*Parent reports*						
P1 Prosocial behavior	−0.33	.742	−2.42	0.48	.634	4.31
P1 Anxiety/depression	0.60	.549	4.77	−0.61	.544	−5.36
P1 ADHD symptoms	0.15	.885	1.11	0.87	.386	7.41
P1 ODD behavior	0.87	.388	6.64	−0.36	.718	−3.18
P1 Aggressive behavior	−0.70	.485	−5.27	−0.38	.707	−3.19
P2 Prosocial behavior	0.40	.691	3.01	0.53	.594	5.10
P2 ODD behavior	0.55	.582	4.23	−0.13	.899	−1.10
P2 Aggressive behavior	−0.52	.601	−3.91	−0.49	.622	−4.07
P3 Prosocial behavior	−0.44	.657	−3.30	0.46	.645	4.21
P3 Anxiety/depression	−0.57	.569	−4.51	0.14	.887	1.26
P3 ADHD symptoms	−0.75	.454	−5.91	0.08	.935	0.68
P3 ODD behavior	−0.04	.965	−0.32	−0.49	.626	−4.56
P3 Aggressive behavior	−1.12	.263	−8.50	−0.28	.778	−2.46
P1 Parenting involvement	−0.21	.835	−1.62	0.80	.425	6.83
P1 Controlling parenting	−0.29	.773	−2.13	−0.54	.592	−4.52
P1 Erratic parenting	−0.72	.475	−5.44	−0.12	.904	−1.00
P1 Corporal punishment	0.56	.575	3.89	0.05	.963	0.41
P2 Parenting involvement	0.41	.683	3.02	0.80	.426	7.00
P2 Controlling parenting	−0.59	.554	−4.48	−0.25	.801	−2.08
P2 Erratic parenting	0.42	.678	3.16	0.15	.884	1.28
P2 Corporal punishment	0.60	.549	4.36	−0.04	.972	−0.31
P3 Parenting involvement	0.37	.709	2.80	0.40	.688	3.56
P3 Controlling parenting	−1.08	.281	−7.63	−0.41	.686	−3.47
P3 Erratic parenting	−0.31	.756	−2.43	−0.17	.866	−1.48
P3 Corporal punishment	0.09	.930	0.64	−1.10	.274	−9.75
P2 Child’s attitude toward homework	0.44	.662	3.32	0.00	1	0.00
P2 Teacher–student relationship	−0.04	.971	−0.29	−0.89	.372	−7.95

S—student, T—teacher, P—parent; the numbers next to S, T and P indicate the period of measurement, from wave 1 to wave 3 when the students were 7, 8 and 9 years old, respectively; Bull Vict—bullying victimization; Bull Perp—bullying perpetration. In order to check the inferences reached from the *t* tests, we also assessed the quality of the balance using the standardized mean difference (SMD) recommended by Rosenbaum and Rubin (1985), Snodgrasse et al. (2011). A covariate is considered balanced if |*SMD*| < .20

## Appendix 2

See Table 7.

**Table 7:** Post-match assessment of the balance on 106 variables (including teacher gender) between the groups of students with a “more positive” (“high dose”/“treated”) vs. “less positive” (“low dose”/“controls”) relationship with their teachers based on teacher and student reports of the relationship

	Teacher–student relationship					
	Teacher-reported			Student-reported		
	*t*	*p*	*SMD*	*t*	*p*	*SMD*
Student gender	−0.75	.453	−5.39	−0.43	.671	−3.79
Teacher gender	0.18	.860	1.26	−0.95	.342	−9.70
Foreign born status	−0.56	.577	−4.33	0.93	.352	9.00
Single parent home	−0.10	.918	−0.81	0.62	.536	6.05
Parental education	0.63	.528	4.81	0.64	.522	6.67
Special education	0.00	1	0.00	−0.24	.809	−2.40
Socio-economic status	0.79	.430	5.78	−0.56	.574	−5.74
Received PATHS	0.16	.875	1.20	0.40	.690	3.77
Received Triple P	−0.39	.694	−3.03	−1.32	.188	−12.63
*Student reports*						
S1 Prosocial behavior	−0.74	.459	−5.76	−0.35	.726	−3.26
S1 Anxiety/depression	−0.73	.465	−5.72	−0.55	.585	−5.42
S1 ADHD symptoms	−0.20	.841	−1.60	−0.02	.986	−0.17
S1 ODD behavior	0.06	.952	0.47	0.10	.924	0.95
S1 Aggressive behavior	0.00	.997	−0.03	−0.45	.653	−4.15
S2 Prosocial behavior	0.03	.979	0.21	−0.32	.751	−3.02
S2 ODD behaviors	−0.14	.890	−1.03	0.39	.700	3.62
S2 Aggressive behavior	−0.98	.329	−6.91	−0.34	.735	−3.09
S3 Prosocial behavior	−0.63	.527	−4.87	−0.94	.346	−9.41
S3 Anxiety/depression	−1.22	.223	−9.07	−0.78	.438	−7.43
S3 ADHD symptoms	−0.28	.777	−2.17	0.50	.621	4.59
S3 ODD behavior	0.36	.722	2.65	0.54	.591	5.00
S3 Aggressive behavior	0.54	.593	4.11	0.05	.959	0.47
S2 Bull Vict—teasing	−0.78	.437	−6.06	−0.08	.934	−0.79
S2 Bull Vict—steal/destroy	−0.40	.691	−3.20	−0.11	.911	−1.12
S2 Bull Vict—physical	−0.37	.712	−2.85	−0.82	.414	−7.81
S2 Bull Vict—rejection	0.19	.847	1.49	−0.64	.524	−5.89
S2 Bull Perp—teasing	−0.71	.478	−5.44	0.00	1	0.00
S2 Bull Perp—steal/destroy	0.08	.936	0.62	0.36	.718	3.50
S2 Bull Perp—physical	0.09	.927	0.70	−0.21	.833	−2.08
S2 Bull Perp—rejection	0.10	.921	0.76	−0.36	.718	−3.42
S2 Likes school	−1.73	.085	−13.02	−0.34	.736	−3.40
S3 Likes school	0.49	.628	3.75	0.20	.838	1.98
S3 Getting along with peers	−0.87	.386	−6.54	−0.47	.643	−4.37
S3 Teacher–student relationship	0.55	.583	4.31	1.24	.218	11.69
*Teacher reports*						
T1 Prosocial behavior	−0.24	.810	−1.76	−0.82	.411	−7.42
T1 Anxiety/depression	−0.06	.954	−0.43	0.84	.401	8.24
T1 ADHD symptoms	0.66	.512	4.74	−0.15	.884	−1.43
T1 ODD behavior	0.17	.864	1.24	−0.39	.694	−3.64
T1 Aggressive behavior	0.10	.919	0.77	−0.56	.578	−5.07
T1 Non-aggressive problem behavior	0.39	.697	2.69	−0.05	.960	−0.47
T2 Prosocial behavior	−0.20	.844	−1.50	−0.76	.451	−7.16
T2 Anxiety/depression	1.29	.199	9.18	0.63	.527	6.16
T2 ADHD symptoms	0.05	.961	0.35	0.14	.889	1.34
T2 ODD behavior	−0.31	.761	−2.12	0.90	.367	8.56
T2 Aggressive behavior	−0.58	.561	−4.22	0.03	.979	0.25
T2 Non-aggressive problem behavior	−0.26	.799	−1.81	0.80	.425	7.68
T3 Prosocial behavior	−0.44	.664	−3.31	−0.70	.486	−6.22
T3 Anxiety/depression	0.27	.786	2.08	0.44	.658	4.21
T3 ADHD symptoms	0.59	.555	4.44	0.14	.886	1.31
T3 ODD behavior	−0.13	.896	−0.97	0.44	.661	4.15
T3 Aggressive behavior	−0.03	.977	−0.22	0.39	.695	3.64
T3 Non-aggressive problem behavior	−0.02	.982	−0.17	0.84	.400	7.84
T1 Academic—math	−0.46	.646	−3.43	−0.28	.777	−2.78
T1 Academic—language	0.03	.973	0.26	−0.09	.929	−0.87
T1 Academic—motivation	−0.68	.495	−5.10	−0.05	.961	−0.50
T1 Child’s social role—popular	0.30	.762	2.20	0.10	.919	0.95
T1 Child’s social role—rejected	−0.76	.450	−5.82	0.50	.619	4.73
T1 Child’s social role—isolated	−0.35	.724	−2.50	0.68	.497	6.60
T1 Child’s social role—dominant	0.39	.699	3.04	−0.32	.753	−3.02
T2 Academic—math	−1.21	.226	−9.22	−0.88	.380	−8.46
T2 Academic—language	−0.62	.537	−4.61	−0.70	.485	−6.86
T2 Academic—motivation	−1.68	.093	−12.16	−0.78	.436	−7.26
T2 Child’s social role—popular	0.62	.537	4.62	−0.99	.323	−9.23
T2 Child’s social role—rejected	−0.44	.659	−3.38	0.26	.794	2.47
T2 Child’s social role—isolated	−0.48	.631	−3.73	0.54	.591	5.37
T2 Child’s social role—dominant	−0.28	.779	−2.04	−0.27	.788	−2.69
T3 Academic—math	−0.55	.585	−4.14	−0.33	.744	−3.07
T3 Academic—language	−0.45	.656	−3.35	−0.43	.669	−4.08
T3 Academic—motivation	−0.36	.717	−2.77	−0.30	.768	−2.84
T3 Child’s social role—popular	0.49	.626	3.74	−0.05	.958	−0.50
T3 Child’s social role—rejected	−0.31	.757	−2.37	0.79	.431	8.10
T3 Child’s social role—isolated	−0.53	.594	−4.12	0.78	.434	7.76
T3 Child’s social role—dominant	−0.41	.679	−3.27	0.00	1	0.00
T3 Parent interested in school	0.74	.461	5.34	0.13	0.895	1.20
T3 Parent supports school	0.62	.533	4.54	−0.55	.582	−5.58
T3 Teacher–student relationship	0.57	.572	4.05	−0.87	.386	−8.64
T1 School cohesion	−0.38	.703	−2.79	0.55	.584	5.14
T2 School cohesion	0.33	.742	2.35	0.34	.731	3.46
T3 School cohesion	0.54	.589	4.03	0.93	.352	8.39
*Parent reports*						
P1 Prosocial behavior	−1.00	.317	−7.51	−0.22	.825	−2.20
P1 Anxiety/depression	0.33	.741	2.49	0.39	.700	3.84
P1 ADHD symptoms	0.67	.506	5.18	0.44	.659	4.46
P1 ODD behavior	1.80	.072	13.92	0.55	.583	5.62
P1 Aggressive behavior	0.98	.330	7.68	−0.08	.940	−0.75
P2 Prosocial behavior	−0.64	.524	−4.80	−0.02	.982	−0.23
P2 ODD behavior	0.45	.655	3.35	0.48	.630	4.86
P2 Aggressive behavior	0.88	.381	6.76	0.23	.822	2.19
P3 Prosocial behavior	0.11	.909	0.87	0.12	.907	1.19
P3 Anxiety/depression	0.86	.388	6.81	0.60	.548	6.00
P3 ADHD symptoms	0.66	.512	5.12	0.73	.465	7.67
P3 ODD behavior	0.87	.388	6.88	0.43	.667	4.36
P3 Aggressive behavior	0.72	.471	5.59	0.07	.948	0.67
P1 Parenting involvement	0.60	.552	4.32	−0.17	.863	−1.66
P1 Controlling parenting	0.46	.644	3.49	−0.29	.775	−2.87
P1 Erratic parenting	0.72	.472	5.39	−0.21	.836	−1.99
P1 Corporal punishment	0.67	.503	5.26	0.23	.822	2.22
P2 Parenting involvement	0.86	.391	6.52	0.46	.643	4.46
P2 Controlling parenting	0.53	.600	3.97	0.33	.742	3.27
P2 Erratic parenting	1.16	.247	8.68	−0.72	.471	−7.15
P2 Corporal punishment	0.23	.821	1.78	0.18	.857	1.85
P3 Parenting involvement	−0.29	.775	−2.19	0.03	.977	0.27
P3 Controlling parenting	0.84	.401	6.31	−0.09	.932	−0.86
P3 Erratic parenting	0.63	.530	4.81	−0.44	.663	−4.18
P3 Corporal punishment	1.09	.275	8.66	0.07	.942	0.72
P2 Child’s attitude toward homework	−0.71	.480	−5.19	0.08	.939	0.71
P2 Teacher–student relationship	−1.22	.224	−9.61	−0.75	.456	−7.01

S—student, T—teacher, P—parent; the numbers next to S, T and P indicate the period of measurement, from wave 1 to wave 3 when the students were 7, 8 and 9 years old, respectively; Bull Vict—bullying victimization; Bull Perp—bullying perpetration. A covariate is considered balanced if |*SMD*| < .20
